# Wisdom of the silicon crowd: LLM ensemble prediction capabilities rival human crowd accuracy

**DOI:** 10.1126/sciadv.adp1528

**Published:** 2024-11-08

**Authors:** Philipp Schoenegger, Indre Tuminauskaite, Peter S. Park, Rafael Valdece Sousa Bastos, Philip E. Tetlock

**Affiliations:** ^1^ Department of Management, London School of Economics and Political Science, Houghton Street, London WC2A 2AE, UK.; ^2^ Independent researcher, London, UK.; ^3^ Department of Physics, Massachusetts Institute of Technology, 77 Massachusetts Avenue, Cambridge, MA 02139, USA.; ^4^ Department of Psychology, Universidade São Francisco, Av. São Francisco de Assis, 218 - Jardim Sao Jose, Bragança Paulista-SP 12916-900, Brazil.; ^5^ Department of Psychology, University of Pennsylvania, 3720 Walnut St, Philadelphia, PA 19104, USA.; ^6^ The Wharton School, University of Pennsylvania, 3733 Spruce St, Philadelphia, PA 19104, USA.

## Abstract

Human forecasting accuracy improves through the “wisdom of the crowd” effect, in which aggregated predictions tend to outperform individual ones. Past research suggests that individual large language models (LLMs) tend to underperform compared to human crowd aggregates. We simulate a wisdom of the crowd effect with LLMs. Specifically, we use an ensemble of 12 LLMs to make probabilistic predictions about 31 binary questions, comparing them with those made by 925 human forecasters in a 3-month tournament. We show that the LLM crowd outperforms a no-information benchmark and is statistically indistinguishable from the human crowd. We also observe human-like biases, such as the acquiescence bias. In another study, we find that LLM predictions (of GPT-4 and Claude 2) improve when exposed to the median human prediction, increasing accuracy by 17 to 28%. However, simply averaging human and machine forecasts yields more accurate results. Our findings suggest that LLM predictions can rival the human crowd’s forecasting accuracy through simple aggregation.

## Teaser

An ensemble of 12 LLM models achieved forecasting accuracy statistically indistinguishable from that of the human crowd.

## INTRODUCTION

In the field of artificial intelligence (AI), the rapidly increasing capabilities of large language models (LLMs) have shown promise and market competitiveness in a rapidly increasing number of economically valuable and cognitively demanding tasks (*1*, *2*). State-of-the-art LLMs with billions of parameters, built on the transformer architecture (*3*), are trained on a very large amount of internet text data (*4*), before being fine-tuned to enhance their performance on specific tasks and improve their applicability in diverse domains. The LLMs are trained on these data to predict the next word or subword (token) when given an input string. This step of next-token prediction—when applied repeatedly—generates a sequence of tokens that form an output string coherently text-completing the input, often at a level of coherence previously thought to be only achievable by human cognition (*5*–*8*) and at a high level of applicability to chat interfaces and various other settings.

This general training objective of next-token prediction, coupled with fine-tuning, also indirectly results in these LLMs displaying an array of specialized skills, which are often only emergently observed after the fact: in ways that were not—and for all practical purposes, likely could not have been—predicted before the first observation of the given capability (*9*). Such skills include but are not limited to reading comprehension (*10*), strategy (*11*), abstract object classification (*12*), and social science applications (*13*, *14*).

When evaluating the capabilities of a given AI system, the predominant method is to measure how well an AI system performs at fixed benchmarks for specific tasks (*15*). The sizeable advancements achieved by transformer-based LLMs in these domains have rendered many previously established benchmarks obsolete (*16*, *17*), moving the metaphorical goalposts forward in the form of more challenging and comprehensive benchmarks (*18*). It is plausible that a sizeable portion of the unprecedented successes that state-of-the-art LLMs have achieved on past task benchmarks is genuinely due to a deep understanding of the task-relevant cognitive skills (*19*). This argument is corroborated by the economic competitiveness—and even promises of economic superiority—that LLMs are achieving for an increasing array of human occupations (*2*), such as transcription (*20*), translation (*21*), and programming (*19*).

However, it is also plausible that a sizeable portion of LLMs’ successes on task benchmarks is due to a superficial memorization of the task’s solutions and a shallow understanding of training set patterns in general (*22*–*25*). Distinguishing between deep understanding and shallow memorization is a complex challenge and is central to accurately assessing AI systems’ advanced reasoning capabilities. This is akin to the examiner’s problem of testing their student for a deep understanding of the course material, even when many of the potential exam questions can be correctly answered by shallow memorization instead. Just like the student can memorize the answers to exam questions if they see them beforehand, so too can an LLM if its training data contain the questions and answers used in the task benchmark. To resolve this ambiguity, one can exploit the testable presence or absence of the ability to generalize out of distribution: to apply learned knowledge beyond the settings represented in the training data (*26*). Such a test is arguably key to discerning a deep understanding of the task at hand (*27*) but is difficult to design when assessing broad LLM capabilities.

In contrast to task benchmarks, where questions and answers are fixed and potentially contained in an LLM’s training data, there are contexts where this concern can be ruled out fully, for example, when predicting the future in real-world settings (*28*, *29*). This test stands out for its high external validity, in that the correct answer to a given real-world forecasting question cannot be in a given LLM’s training set, as not even the human evaluator knows the answer at the time of data collection. Moreover, the practice of forecasting is omnipresent in the cognitive tasks undertaken by humans, encompassing a wide range of applications from forecasting the trajectory of current events to setting long-term plans. The ubiquity of forecasting—especially in white-collar occupations where the increasing capabilities of LLMs are predicted to disrupt or even replace human professionals (*30*–*32*)—combined with the intrinsic external validity makes testing LLMs’ forecasting capability ideal for assessing their real-world applicability.

One context where LLMs’ forecasting capability can be tested directly is forecasting tournaments. In these tournaments, participants make probabilistic predictions about future occurrences and are then evaluated and rewarded for their accuracy (*33*). The accuracy of these forecasts across a set of questions determines the amount of reputational or monetary reward. Since more precise predictions yield greater rewards, forecasters are incentivized to conduct substantively helpful research and to provide well-informed predictions. The aggregate of these predictions across the crowd of human forecasters is a gold standard for human intelligence gathering. The effectiveness of aggregating competitive forecasts relies on the “wisdom of the crowd” phenomenon: the effect in which the collective accuracy of many forecasters’ predictions often surpasses that of any one individual among the crowd. This concept is supported by extensive research across various fields such as prediction markets (*34*) and political forecasting (*35*), showing that the combined forecasts of many individuals tend to be precise (*36*–*38*). This wisdom of the crowd effect relies on independent and unbiased judgments, which achieves an error-cancelation effect (*39*, *40*). As Budescu (*41*) pointed out, this aggregation mechanism increases information and accounts for extremes, with the wisdom of the crowd effect also holding in contexts of biased inputs (*42*) or when there are correlations among judgments (*40*), showing remarkable robustness. There is a large literature on how to improve this aggregation process (*43*–*45*), with a central takeaway being that a simple median is an unexpectedly powerful aggregation mechanism: one that works across a myriad of contexts.

Past work has compared the prediction performance of frontier models against a human crowd. With respect to evaluating a single model, Schoenegger and Park (*28*) found that the frontier model GPT-4 performed poorly when comparing its predictions to that of a crowd drawn from a forecasting tournament. GPT-4 did not even significantly outperform the no-information benchmark strategy of predicting 50% on every question. Also, Halawi *et al*. (*46*) investigated the prediction capabilities of an LLM system, including a combination of news retrieval and reasoning systems. They replicated the finding of Schoenegger and Park that individual models not only show poor prediction accuracy but also found that their optimized system approached the accuracy of the human crowd. This suggests that individual LLMs may have poor forecasting accuracy but can produce accurate predictions if they are set in an advanced system.

A hypothesis worth probing is that the underperformance of individual LLMs in real-time forecasting compared to human crowds may, at least in part, be due to not making use of the wisdom of the crowd effect. It may thus be the case that LLMs’ forecasting accuracy may be able to reach human-level performance if machine predictions are sampled from a wide set of diverse models. To test this question, we simulate one such crowd of diverse LLMs and draw questions from a real-world forecasting tournament. We directly compare the LLM crowd’s estimate to that of the human crowd, without introducing further additions like retrieval systems.

In Study 1, we test this LLM ensemble approach. Specifically, we aggregate 12 LLMs’ forecasts into a collective crowd forecast, leveraging the diversity inherent in the different models’ training data, parameters, and methodologies (such as idiosyncratic fine-tuning). We first test whether the LLM ensemble, unlike GPT-4 in the study of Schoenegger and Park (*28*), will significantly outperform the no-information benchmark in a forecasting tournament. This benchmark provides a minimal benchmark of accuracy that is equivalent to guessing 50% on every question.

**Null hypothesis 1, Study 1**: The average of median LLM forecasts is neither statistically significantly more nor less accurate than the 50% baseline, $H_{0_{1}}:{\bar{B}}_{\text{LLM}}=0.25$.

We also conduct a stronger test of whether the LLM ensemble will significantly outperform the human crowd drawn from the real-world forecasting tournament. For both studies, we use a 3-month tournament run on the platform Metaculus as our human crowd comparison. This provides a more direct comparison of the two aggregated forecasts and would present a result that has not been achieved so far.

**Null hypothesis 2, Study 1**: The average of median LLM forecasts is neither statistically significantly more nor less accurate than the average of median human forecasts, *H*_0_2__ : μ_LLM_ = μ_Human_.

Last, for Study 1, we test for differences in forecasting accuracy between the 12 models. Some of these models are variations of each other, like GPT-4 versus GPT-4 with Bing access, PaLM2 versus PaLM2 in Bard, and Llama-2-70B versus Solar-0-70B, while others differ on more fundamental grounds (e.g., parameter counts and open versus closed source). Testing for statistically significant differences between models with different capabilities, endpoints, fine-tunings, sizes, etc., might provide further insight into which aspects help and which aspects hinder prediction accuracy.

**Null hypothesis 3, Study 1**: There are no statistically significant differences in the average accuracy across the different LLMs and humans, *H*_0_3__ : μ_1_ = μ_2_ = … = μ*_k_*.

In Study 2, we investigate the ability of two frontier models (GPT-4 and Claude 2) to integrate human intelligence into their forecast updating. This contributes to the literature on human-AI interactions. While previous work has focused on how AI can improve human predictions (*29*), our study looks at the reverse: how human forecasts can improve LLM predictions. This is studied in a context where models update their forecasts in response to receiving the human crowd’s prediction. This investigation of updating behavior is grounded in the premise that access to external information, such as the median forecast of a human crowd, can serve as a valuable reference point for recalibrating predictions. The interaction between human and machine intelligence in this context is of particular interest, as it exemplifies the potential synergies that can emerge from integrating the intuitive, experience-based judgments of humans with the data-processing capabilities of LLMs.

We first investigate whether, for each of the two frontier LLMs, its average forecast becomes more accurate after being presented with the human crowd’s median forecast. This is arguably the most straightforward test of whether human cognitive output in this setting can augment machine-generated forecasts, as measured by forecasting accuracy.

**Null hypothesis 1, Study 2**: There is no statistically significant difference in the average accuracy of either LLM before and after having been provided the human crowd median, *H*_0_1__ : μ_pre_ = μ_post_.

We next investigate the impact that exposure to the human median forecast has on the precision of LLM forecasts. Specifically, we investigate whether the prediction intervals become narrower, indicating increased confidence in the forecasts: an effect that would suggest that LLMs can extract nontrivial information value from the human median forecast.

**Null hypothesis 2, Study 2**: The size of the prediction intervals does not become narrower after exposure to the human crowd median, *H*_0_2__ : Δ_range_ ≥ 0.

Last, we investigate the relationship between the initial deviation of LLM forecasts from the human median and the magnitude of subsequent adjustments. This probes the extent to which larger discrepancies prompt more substantial forecast revisions, as would be expected.

**Null hypothesis 3, Study 2**: The magnitude of LLM forecast adjustments is not correlated with the initial deviation of their forecasts from the human crowd median, *H*_0_3__ : ρ = 0.

Both studies advance the literature on LLM prediction capabilities. Building on the past work of Schoenegger and Park (*28*), the present paper examines an LLM ensemble approach instead of a single model. In addition, while Schoenegger *et al*. (*29*) have looked at how AI predictions can improve human accuracy, the present paper tests the converse, thereby helping complete the picture of how humans and AI systems may interact in real-world contexts that require accurate forecasting. This overall adds to a growing literature on AI and judgemental forecasting (*46*).

## METHODS

All analyses were preregistered on the Open Science Framework, as detailed in https://osf.io/sb6mw/?view_only=395ab8faccba419c91f5f12dcaf97ce6. We clearly label all exploratory and non-preregistered analyses as such throughout the paper to indicate which tests were decided on after having seen the data.

### Study 1

In Study 1, we collected data from a total of 12 diverse LLMs to simulate the LLM crowd. To have a broad set of models, we tried to vary the size of the models, the access to additional tools (like internet search), whether they were open source, and the company’s country. Specifically, these 12 models were GPT-4, GPT-4 with Bing, Claude 2, GPT3.5-Turbo-Instruct, Solar-0-70b, Llama-2-70b, PaLM 2 (Chat-Bison@002), Coral (Command), Mistral-7B-Instruct, Bard (PaLM 2), Falcon-180B, and Qwen-7B-Chat. We accessed each model through a web interface and did not query any models via their application programming interfaces (APIs) to hold the query method constant, as not all models had API access at the time. This resulted in the use of default parameters (e.g., temperature) for all models. These web interfaces included company-specific interfaces like those offered by OpenAI, Anthropic, Cohere, and Google for their respective models, as well as interfaces provided by other third parties such as Poe, Hugging Face, and ModelScope that provided access to the remaining models. We took this approach to maximize the number of models for which we could reliably query throughout the study’s data collection period while retaining heterogeneity of model specifications, as our goal was to draw on a diverse set of models. In addition, this also kept our study in the context of publicly available and easily accessible models, making it easier to implement this approach with a low amount of resources and effort. Our final set of models includes frontier proprietary models (GPT-4 and Claude 2) and open-source models (e.g., Llama-2-70b and Mistral 7B-Instruct) from a variety of demographically diverse companies originating from China, France, the United Arab Emirates, South Korea, Canada, and the United States. We also have a variety of models with internet access (e.g., GPT-4 with Bing, Bard, and Coral) and a large diversity of model sizes, ranging from 7 billion parameters to an estimated 1.6 trillion. We monitored updates to the original models at the web interfaces and responded as follows to changes: In response to the release of GPT-4-Turbo, on 6 November, we queried the “Classic” model instead. For the upgrade to Claude 2.1, we did not switch the query method on 21 November. When Bard switched, at least in part, to Gemini Pro from PaLM 2, we ceased data collection of this model via the Bard interface on 6 December. For a list of all models and their central specifications, see Table 1 below.

**Table 1.** Detailed characteristics of each LLM tested.

Model	Company	Internet access	Open source	Hosting platform	Country of company
GPT-4	OpenAI	No	No	OpenAI	United States
GPT-4 Bing	OpenAI	Yes	No	OpenAI	United States
Claude 2	Anthropic	No	No	Anthropic	United States
GPT-3.5-Turbo-Instruct	OpenAI	No	No	OpenAI	United States
Solar-0-70B	Upstage	No	Yes	Poe	South Korea
Llama-2-70B	Meta	No	Yes	Poe	United States
PaLM 2 (Chat-Bison@002)	Google	No	No	Poe	United States
Coral (Command)	Cohere	Yes	No	Cohere	Canada
Mistral-7B-Instruct	Mistral	No	Yes	Poe	France
Bard (PaLM 2)	Google	Yes	No	Google	United States
Falcon 180B	Technology Innovation Institute	No	No	Hugging Face	United Arab Emirates
Qwen-7B-Chat	Alibaba Cloud	No	Yes	ModelScope	China

To assess the prediction capabilities of these models, we drew on a set of forecasting questions that were asked in real time at a public forecasting tournament that ran from October 2023 to January 2024 on the platform Metaculus, where over the course of this tournament, 925 human forecasters provided at least one prediction. In this tournament, forecasters were able to sign up with Metaculus (*47*) and predict as many questions as they wanted. The topics of the posed questions ranged from conflict in the Middle East, interest rates, literary prizes, and English electoral politics to Indian air quality, cryptocurrency, consumer technology, and space travel. We focused exclusively on binary probabilistic forecasts, collecting across a total of 31 questions. These questions were crowdsourced and written by forecasters (including one author of this paper). All questions were reviewed by Metaculus staff for quality before opening them up for forecasting. Each question included a question title, a background section detailing the context of the question being asked, and a resolution passage that spelled out how the question would resolve. We drew on the same set of questions and used the publicly available human median predictions for each question as the human benchmark. For a full list of the questions, see table S1.

For every question, within 48 hours of the question opening, we queried each model three independent times and recorded their predictions at the default settings. We recorded both the quantitative forecast and the qualitative rationale for all entries. If a model was unresponsive because of a technical reason, then we attempted to collect a forecast 24 hours after the first failed attempt. If a model failed to provide a forecast for non-technical reasons like model censorship or content restrictions after several attempts, then we did not reattempt data collection and instead recorded the prediction as missing. For each question, we prompted each model three times and recorded all predictions. If a model only responded with “Yes” or “No” as their prediction, then we coded this as 99 and 1%, respectively, though we note that this happened in less than 1% of cases across models. For cases in which a model failed to provide a forecast for the second or third run after having provided a forecast before, we continued to query the model until all three forecasts were provided.

The prompt that we used for all models included instructions on how to format the output and a number of prompting techniques that included instructing the model to respond as a superforecaster, using a persona prompt, and approaching questions via a simple chain-of-thought method. The central motivating factor for deciding on the prompt used in our experiment was not accuracy but the consistency of output across questions and models. The resulting prompt included detailed question background, resolution criteria, and question text as they were posed on the public forecasting tournament; see Box 1.

Box 1. Full prompt Study 1.

In this chat, you are a superforecaster that has a strong track record of accurate forecasts of the future. As an experienced forecaster, you evaluate past data and trends carefully and aim to predict future events as accurately as you can, even though you cannot know the answer. This means you put probabilities on outcomes that you are uncertain about (ranging from 0 to 100%). You aim to provide as accurate predictions as you can, ensuring that they are consistent with how you predict the future to be. You also outline your reasons for this forecasting. In your reasons, you will carefully consider the reasons for and against your probability estimate, you will make use of comparison classes of similar events and probabilities and take into account base rates and past events as well as other forecasts and predictions. In your reasons, you will also consider different perspectives. Once you have written your reasons, ensure that they directly inform your forecast.Then, you will provide me with a number between 0 and 100 (up to two decimal places) that is your best prediction of the event. Take a deep breath and work on this problem step by step.

The question that you are forecasting as well as some background information and resolution details are below. Read them carefully before making your prediction.

**Background:**

**Resolution:**

**Question:**

For every set of machine forecasts, we also recorded the publicly available median human crowd prediction at the end of the day that the machine forecast was entered. If the prediction was entered on the first day, then we collected the human crowd predictions at the end of the second day for which the question was open to allow for higher participation rates. This was done to ensure a fair comparison of machine and human forecasts. Many LLMs can recall the current date, thus making timed forecasts of the nature studied here potentially sensitive to asynchronous queries, while also introducing bias with respect to the human crowd. For roughly half of the questions, the human forecasters were not able to see the human crowd forecast, though there was substantial heterogeneity when the community predictions were made available to human forecasters. In 15 out of 31 questions, our data were collected before the revelation of the community prediction to the human forecasters. Note that this variation in community prediction revelation could not be directly controlled on our end, as this is a decision made by Metaculus based on question author specifications and judgments of public interest, where questions of high public interest have their community prediction revealed earlier than scheduled.

**Fig. 1.**
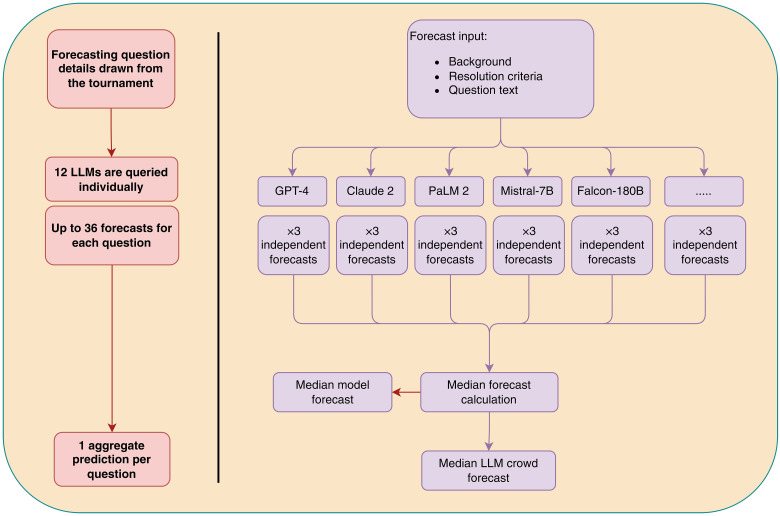
Overview of the LLM ensemble mechanism.

For the human forecasts, we took the publicly available median forecast for each question. For the LLM ensemble approach, we computed the median from all nonmissing forecasts on each question. We also computed the median forecast on each question for each model, to enable cross-model comparisons. See Fig. 1 for an overview of our LLM ensemble approach.

### Study 2

In Study 2, we focused exclusively on two frontier models, GPT-4 and Claude 2. For our study context, we used the same real-world forecasting tournament as in Study 1, functioning as a source of questions and human forecasts. For Study 2, we used a within-model research design that collected two forecasts (pre- and postintervention) per model run for each question. Each question was posed three times at the standard temperature settings, resulting in six forecasts per model for each question. Our goal was to investigate LLM updating behavior with respect to human cognitive output, i.e., whether and how LLMs take into account the human prediction estimates that forecasting tournament aggregates provide. We queried GPT-4 and Claude 2 via the OpenAI and Anthropic websites, respectively.

Note that for Study 2, we had to wait until all relevant community predictions were made publicly available. This means that compared to Study 1, there was substantial heterogeneity with respect to timing and prompting, making accuracy comparisons between the two studies problematic. Hence, while Study 1 was our cleanest attempt at measuring the LLM ensemble accuracy, Study 2 was primarily designed as a measure of directional updating, as opposed to directly comparable accuracy differences.

We used a substantially longer and more elaborate set of prompts than in Study 1. The first prompt built on the “10 commandments of superforecasting” (*48*) and the literature on forecasting and updating, instructing models to carefully distinguish different degrees of doubt, strike the correct balance between under- and overconfidence, and break difficult problems into subproblems that are easier to solve, among other instructions. The second prompt—the intervention—informed the model of the respective human crowd’s median forecast and asked it to update if necessary, as well as to outline the reasons for the update (if any). No additional information was provided, and both models could not access the internet. For the full text of both prompts, see Boxes 2 and 3, respectively.

Box 2. Initial prompt for Study 2.

In this chat, you are a superforecaster who has a strong track record of accurate forecasting. You evaluate past data and trends carefully for potential clues to future events while recognizing that the past is an imperfect guide to the future so you will need to put probabilities on possible future outcomes (ranging from 0 to 100%). Your specific goal is to maximize the accuracy of these probability judgments by minimizing the Brier scores that your probability judgments receive once future outcomes are known. Brier scores have two key components: calibration (across all questions you answer, the probability estimates you assign to possible future outcomes should correspond as closely as possible to the objective frequency with which outcomes occur) and resolution (across all questions, aim to assign higher probabilities to events that occur than to events that do not occur).You outline your reasons for each forecast: list the strongest evidence and arguments for making lower or higher estimates and explain how you balance the evidence to make your own forecast. You begin this analytic process by looking for reference or comparison classes of similar events and grounding your initial estimates in base rates of occurrence (how often do events of this sort occur in situations that look like the present one?). You then adjust that initial estimate in response to the latest news and distinctive features of the present situation, recognizing not only the need for flexible adjustments but also the risks of overadjusting and excessive volatility. Superforecasting requires weighing the risks of opposing errors, e.g., failing to learn from useful historical patterns versus over-relying on misleading patterns. In this process of error balancing, you draw on the 10 commandments of superforecasting (Tetlock & Gardner, 2015) and on other peer-reviewed research on superforecasting:

1. Triage.

2. Break seemingly intractable problems into tractable subproblems.

3. Strike the right balance between inside and outside views.

4. Strike the right balance between under- and overreacting to evidence.

5. Look for the clashing causal forces at work in each problem.

6. Strive to distinguish as many degrees of doubt as the problem permits but no more.

7. Strike the right balance between under- and overconfidence, between prudence and decisiveness.

8. Look for the errors behind your mistakes but beware of rearview-mirror hindsight biases.

9. Bring out the best in others and let others bring out the best in you.

10. Master the error-balancing bicycle.

Once you have written your reasons, ensure that they directly inform your forecast.

Then, you will provide me with your forecast which is a range between two numbers, each between 0 and 100 (up to two decimal places) that is your best range of prediction of the event. Output your prediction as “My Prediction: Between XX.XX% and YY.YY%”. Take a deep breath and work on this problem step by step.

The question that you are forecasting as well as some background information and resolution criteria are below. Read them carefully before making your prediction.

**Background:**

**Resolution Criteria:**

**Question:**

Box 3. Prediction intervention prompt for Study 2.

You have made your forecast based on careful reasoning and analysis. Now, consider the following new piece of information: The median crowd prediction in the forecasting tournament where this question was posed was XXX%. Please adjust your reasoning and forecast based on this information, as you deem appropriate. The large research literature on the wisdom of the crowd suggests that it is difficult for any single forecaster to out-predict crowd medians or averages. However, there are occasions when the crowd has proven to be wrong. In considering whether/how much to revise your earlier forecast, keep in mind the theme of error balancing: the need to balance the risk of giving too little weight to the crowd judgment versus the risk of over-relying on the crowd. Please explain how you balanced these risks. Please also make this prediction be in the same format as before: “My Prediction: Between XX.XX% and YY.YY%.”

For both prompts, we collected forecasts not as point estimates but as probability ranges between 0 and 100%, with two–decimal point specificity. For further analysis, we treat the midpoint of this range as the point estimate and the provided predictions as upper and lower estimates. The human crowd median provided to the models was collected within 48 hours of the community prediction being revealed, to allow human forecasters to learn about it and update their forecasts accordingly. This generally led to more well-calibrated predictions and as such, a more impactful intervention.

## RESULTS

### Study 1

We collected, across the 31 questions, a total of 1007 individual forecasts from the 12 LLMs that make up the ensemble. For 109 forecasts that we did not collect, this was due to technical problems with the model or interface at the time of forecast collection—in the case of Falcon-180B and PaLM 2—or because other models selectively chose not to answer certain questions, presumably due to their content restriction policies—this was sometimes the case for Coral (Command) and Qwen-7B-Chat. We also recorded some missing forecasts for Bard, which was due to the fact that the underlying model powering the interface was changed to Gemini Pro. To ensure consistency and allow comparisons between the different contexts of PaLM 2, we stopped collecting data at this point.

**Fig. 2.**
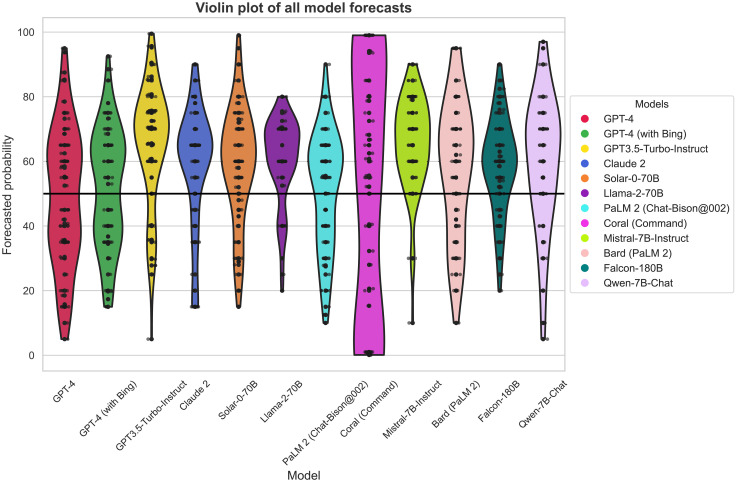
Violin plot of all LLM predictions across all questions.

Across all models and questions, we observe a minimum raw forecast value of 0.1% and a maximum raw forecast value of 99.5%, with a median forecast of 60%. This indicates that the LLMs are more likely to make predictions above the 50% midpoint, with the mean forecast value of the crowd *M* = 57.35 (SD = 20.93) being significantly above the 50% mark, *t*(1006) = 86.20, *P* < 0.001. The total question set resolved close to evenly, with 14 out of 31 questions resolving positively. This imbalance thus suggests that LLM predictions generally favor positive resolutions above and beyond the appropriate empirical expectation, with just more than 45% of questions resolving positively. Such a bias toward more positive predictions may be a function of the machine equivalent of acquiescence bias (*49*), where human responders tend to favor the positive/agreement option irrespective of question content (*50*). This is one aspect in which we observe human forecaster behavior in our machine predictions that may prove useful avenues for improvements in accuracy going forward. See Fig. 2 for a violin plot of all model forecasts across all questions that shows heterogeneity between models of forecast distribution, ranges, and acquiescence bias.

To assess forecasting accuracy, we use the strictly proper scoring rule (*51*) of Brier scores (*52*), a scoring metric that assesses the accuracy of probabilistic predictions by taking the mean squared difference between the forecasted probability and the actual outcome. Because we are not interested in binary classifications or decisions, the Brier score’s ability not only to assess whether the outcome was correct in a binary setting but also to determine how accurate the probabilistic predictions were makes it the preferred measure of accuracy. It is defined mathematically as

$$\text{Brier Score}={\left(f_{i}-o_{i}\right)}^{2}$$

where *f_i_* is the forecasted probability for the instance, and *o_i_* is the actual outcome, which can be 0 or 1. A lower Brier score indicates higher accuracy, with 0 being the perfect accuracy score. A score of 0.250 represents a typical benchmark that would be arrived at if all predictions were 50%.

Testing our first hypothesis as preregistered, we investigate whether the LLM crowd can outperform the simple baseline of assigning a 50% prediction on every question, a baseline that GPT-4 was unable to beat in previous work (*28*). To arrive at the notion of aggregate forecast that will be used for this analysis and further analyses, we calculate the median LLM forecast across all models for every question. We then take these medians and average them across all questions. Next, we compare this average to a Brier score of 0.25 (the result of predicting 50% on all questions). We can reject our null hypothesis, with the LLM crowd, *M* = 0.20 (SD = 0.12), being significantly more accurate than the benchmark, *t*(30) =−2.35, *P* = 0.026. This is evidence that crowd-aggregated LLM forecasts can improve upon basic benchmarks and provide valuable probabilistic predictions.

Next, we compare the LLM crowd’s performance to that of the human crowd for our second and most central hypothesis, directly putting the two crowd-aggregation mechanisms head-to-head. To do this, we use the same LLM-crowd average as before (taking the median LLM prediction on each question and averaging up the Brier scores across questions). We compare this to the average of the median human predictions on the same questions. More detailed distributional statistics are presented in table S2.

**Fig. 3.**
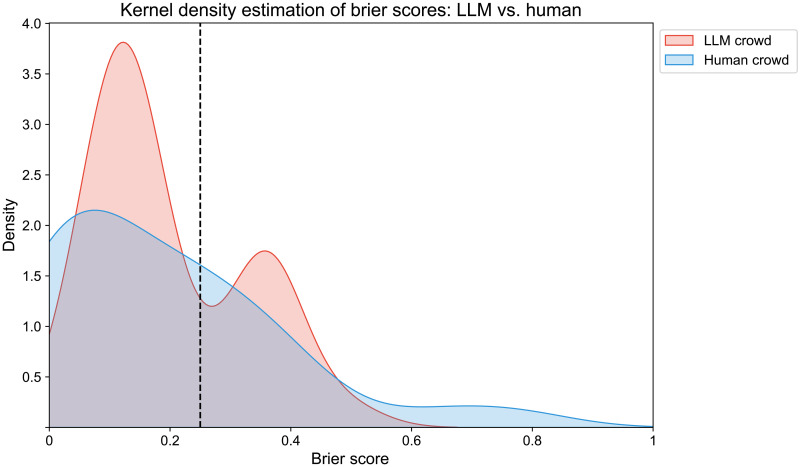
Kernel density estimate of the LLM-crowd and human-crowd forecasts (averaged median scores over all questions). Vertical dotted black line represents the 50% baseline.

In our preregistered analysis, we fail to find statistically significant differences between the LLM crowd’s mean Brier score of *M* = 0.20 (SD = 0.12) and that of the human crowd, *M* = 0.19 (SD = 0.19), *t*(60) = 0.19, *P* = 0.850. See Fig. 3 for a kernel density estimate of the LLM-crowd and human crowd forecasts.

This result only enables us to directly conclude that the LLM crowd is neither more nor less accurate than the human crowd in the question set studied here. To provide some evidence in favor of the equivalence of these two approaches, we conduct a non-preregistered equivalence test with the conventional medium effect size of Cohen’s *d* = 0.5 corresponding to the equivalence bounds (*53*), which allows us to test whether the effect is zero or less than a 0.081 change in Brier scores. For these equivalence bounds, we find that the LLM crowd and the human crowd are equally accurate, with both tests for the lower bound, *t*(60) = 2.16, *P* = 0.017, and the upper bound, *t*(60) = −1.78, *P* = 0.040, being statistically significant. This provides evidence that the LLM crowd is as accurate as the human crowd within these bounds. However, it is important to note that bounds of 0.08 in Brier scores are wide, suggesting that we are unable to identify a narrow null effect in the results provided here.

**Fig. 4.**
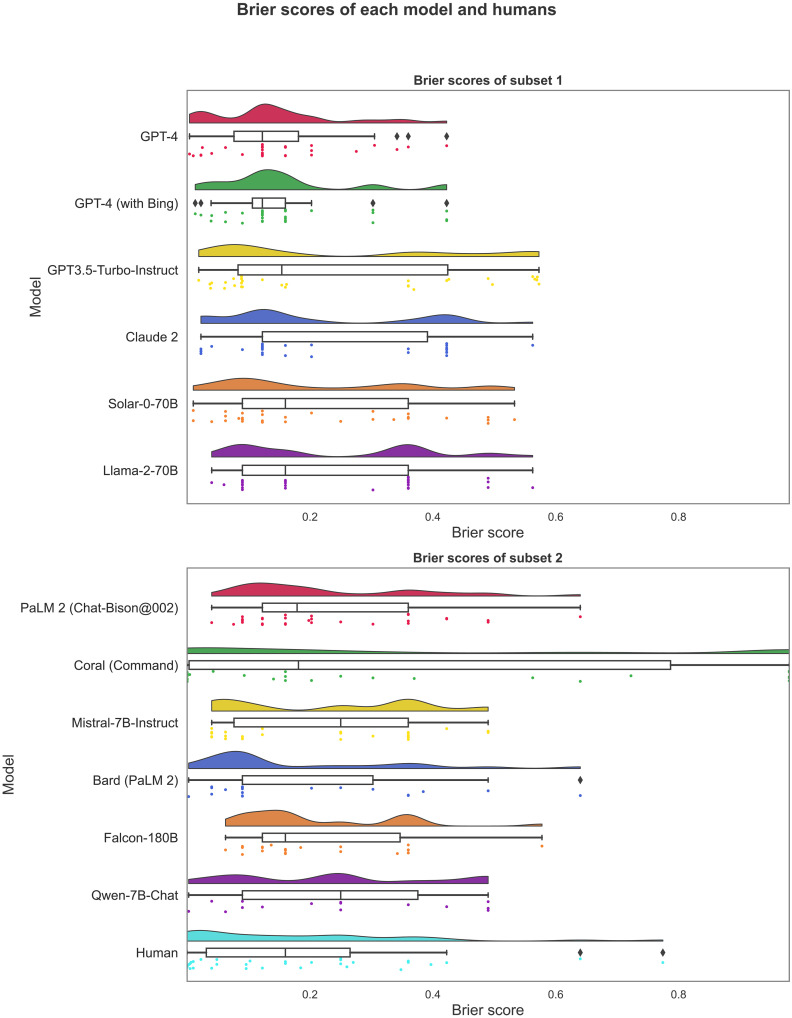
Raincloud plots of Brier scores for each LLM and for the human crowd.

For our third null hypothesis, we compare the forecasting accuracy of each model (and of the human crowd) against each other to find potential effects of internet access (GPT-4 versus GPT-4 with Bing) or access points (Bard with PaLM2 versus PaLM2). Using an analysis of variance, we find significant aggregate differences, *F*(12,354) = 2.64, *P* = 0.002, leading us to reject our third null hypothesis. Using Tukey’s post hoc test to adjust for multiple comparisons in the post hoc pairwise tests, we find that Coral (Command) underperforms a set of models (e.g., Claude 2 and GPT-4) as well as the human crowd. However, we fail to find statistically significant effects between any other pairs not involving Coral (Command), thus being unable to provide evidence in favor of or against potential effects of internet access, access points, or fine-tuning on prediction accuracy. See Fig. 4 for raincloud plots of the LLMs’ Brier scores as well as those of the human crowd. Also, see Table 2 for the models’ average Brier scores, where we find that the aggregate’s Brier score of 0.20 is numerically lower than 9 out of the 12 individual models. However, this difference is not statistically significant after adjustments; see table S3. In further non-preregistered analyses, we provide a conservative test of the “wisdom of the silicon crowd,” comparing all forecasts of the ensemble medians (of model medians) with the model predictions. We find that for 19 out of 31 questions, the ensemble median is above the 50th percentile, and in eight cases, it is at the 50th percentile; see table S4.

**Table 2.** Average Brier score for each model.

Model	Accuracy	SD
GPT-4	0.15	0.11
GPT-4 (with Bing)	0.16	0.11
Bard (PaLM 2)	0.19	0.17
Falcon-180B	0.21	0.13
Claude 2	0.21	0.16
Solar-0-70B	0.22	0.16
PaLM 2 (Chat-Bison@002)	0.23	0.15
Mistral-7B-Instruct	0.24	0.16
Qwen-7B-Chat	0.24	0.17
GPT3.5-Turbo-Instruct	0.25	0.20
Llama-2-70B	0.25	0.16
Coral (Command)	0.38	0.40
Human	0.19	0.19

For all three hypotheses, we implemented the Benjamini-Hochberg (BH) procedure to adjust the *P* values obtained from multiple hypothesis tests. This method was selected to control the false discovery rate (FDR) and thereby reduce the risk of type I errors. The original *P* values for null hypotheses 1, 2, and 3 were 0.026, 0.850, and 0.002, respectively. These *P* values were first sorted in ascending order and then ranked accordingly. The adjusted *P* values were computed using the BH procedure, which calculates the adjusted *P* value for the *i*th hypothesis as $\text{min}\left\{1,\frac{p_{i}\times m}{i}\right\}$, where *p_i_* is the *i*th *P* value in the sorted list, *m* is the total number of hypotheses tested, and *i* is the rank of the *P* value. The results show that the adjusted *P* values for the hypotheses were 0.039 for the first hypothesis (original *P* = 0.026), 0.850 for the second hypothesis (original *P* = 0.850), and 0.006 for the third hypothesis (original *P* = 0.002). These results indicate that our rejections of the first and third null hypotheses remain robust after adjusting for multiple comparisons.

**Fig. 5.**
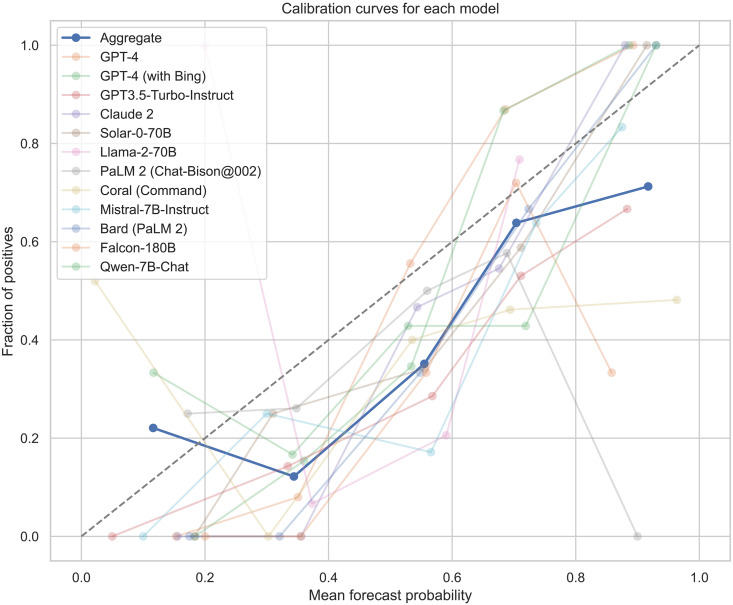
Calibration plot for the LLM aggregate (bolded) and for each comprising LLM (nonbolded).

For further non-preregistered analyses, we conduct calibration analyses using the Murphy decomposition (*54*, *55*) to provide data on how well-calibrated the models are in this context, i.e., how reliably their probability estimates match the fraction of real outcomes. In Fig. 5, calibration curves for each model and their aggregate are plotted against the ideal 45° dotted line. This dotted line represents perfect calibration, where predicted probabilities match observed frequencies. Deviations from this line indicate calibration errors: Curves above the line suggest underconfidence (predicting events as less likely than they actually are), while those below indicate overconfidence (predicting events as more likely than they actually are). Figure 5 visually represents how closely the models’ predictions align with actual outcomes. We also calculate the calibration index (CI) which quantifies this deviation, with lower values indicating better calibration. CI is calculated using the formula

$$\text{CI}=\frac{1}{N}\sum_{k=1}^{K}N_{k}{\left(f_{k}-o_{k}\right)}^{2}$$

where *N* is the total number of forecasts, *K* is the number of bins, *N_k_* is the number of forecasts in bin *k*, *f_k_* is the mean forecast probability in bin *k*, and *o_k_* is the observed relative frequency in bin *k*. This weights each bin’s contribution to the CI by the number of forecasts it contains. This approach ensures that bins with more forecasts, which provide a more statistically reliable estimate of forecasting accuracy, have a proportionately greater impact on the overall CI.

Our results demonstrate poor calibration of most models and overconfidence of the aggregate, suggesting that models overpredict outcomes compared to their actual rate of occurrence; see Fig. 5. This is in line with the finding that we find an acquiescence bias of LLMs on a question set where less than half of questions resolve positively, which is again an effect found in human forecasters as well. We also find generally poor calibration across all models, with some substantial differences in the CI scores; see Table 3. This suggests that a further line of research may build upon improving models’ calibration, in an attempt to improve machine prediction capabilities and reliability further.

**Table 3.** Calibration index values for all LLMs.

Model	Calibration index
Falcon-180B	0.027
Qwen-7B-Chat	0.055
PaLM 2 (Chat-Bison@002)	0.068
Bard (PaLM 2)	0.071
Llama-2-70B	0.071
GPT-4	0.075
Mistral-7B-Instruct	0.080
Solar-0-70B	0.081
Claude 2	0.082
GPT-4 (with Bing)	0.088
GPT3.5-Turbo-Instruct	0.106
Coral (Command)	0.212
Aggregate	0.041

In additional non-preregistered analyses, we also look at potential patterns of accuracy between distinct types of questions. In table S5, we list the LLM crowd’s accuracy on each individual question, splitting questions into different types of topics. While the sample of questions is very low and should be interpreted with great caution, we report the following mean accuracy scores for each topic: Law: 0.100 (*n* = 3), Literature: 0.120 (*n* = 1), Economics: 0.143 (*n* = 4), Conflict: 0.171 (*n* = 7), Technology: 0.173 (*n* = 3), Politics: 0.237 (*n* = 9), Climate: 0.303 (*n* = 3), and Education: 0.360 (*n* = 1). The low number of questions makes rigorous analysis testing for the difference in accuracy between topic areas not possible.

Furthermore, we also report the following additional observations pertaining to our collected data. First, similar to the presence of acquiescence bias discussed above, we also find that model predictions are substantially more likely to be on round numbers than adjacent numbers. For example, across all questions and all models, a total of 38 predictions were entered for 50%, but no predictions were given for 49 or 51%; see table S6. This is an unexpected commonality between human response patterns and model outputs that is in line with the literature on how LLM outputs mirror a number of human biases (*56*–*58*). These data suggest that some of the model outputs might be subject to similar biases that reduce prediction accuracy in humans, hinting at a potential point of improvement for future iterations aimed at increasing model accuracy.

### Study 2

For Study 2, we collected a total of 186 primary forecasts and 186 updated forecasts from both frontier models (GPT-4 and Claude 2) over the 31 binary questions studied. Neither model refused to provide a forecast or failed to respond to our queries.

First, we test whether exposure to the human crowd median improves model accuracy. We can reject the first null hypothesis of Study 2 for both models: For GPT-4, there is a statistically significant difference in Brier scores before and after exposure to the human median, with an average Brier score for the primary forecast of 0.17 (SD: 0.13) and an updated score of 0.14 (SD: 0.11), *P* = 0.003. For Claude 2, we also find a statistically significant difference in Brier scores before and after exposure to the human median, improving from 0.22 (SD: 0.19) to 0.15 (SD: 0.14), *P* < 0.001. This suggests that the provision of human cognition in the form of crowd forecasts can improve model prediction capabilities.

**Fig. 6.**
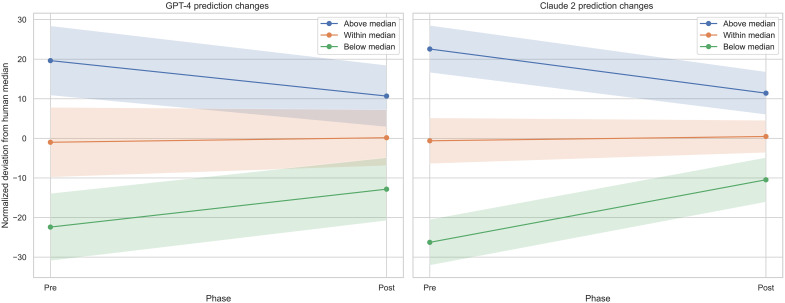
LLM forecasts for GPT-4 (left) and Claude 2 (right) before and after exposure to the human forecast. Colors distinguish first forecasts above, below, or within 20 percentage points of the human median forecast. Highlighted changes and intervals are of the respective median forecast within that group.

We also find that when testing our second hypothesis, the size of the prediction interval narrows after exposure to human crowd predictions that lie within the probability range provided by the model, as would be predicted by theory. The prediction intervals for GPT-4 become significantly narrower after exposure to the human median, ranging from an average interval size of 17.75 (SD: 5.66) to 14.22 (SD: 5.97), *P* < 0.001. The prediction intervals for Claude 2 also become significantly narrower after exposure to the human median forecast, narrowing from 11.67 (SD: 4.201) to 8.28 (SD: 3.63), *P* < 0.001. This suggests that the models appropriately reduce their prediction uncertainty due to their incorporation of the additional information contained in the human forecasts. See Fig. 6 for a graphical illustration of LLM forecasts for each of the two models tested, before and after exposure to the human forecasts.

Last, with respect to our third hypothesis, we analyze whether LLMs’ updates are proportional to the distance between their point forecast and that of the human benchmark. We can reject our null hypothesis for both models, finding a significant correlation between the initial deviation and the magnitude of forecast adjustment for GPT-4, *r* = 0.88, *P* < 0.001; as well as for Claude 2, *r* = 0.87, *P* < 0.001. This suggests that models move their predictions roughly in accordance with how distant their prediction is from the human median.

As in Study 1, we use the BH procedure for controlling multiple comparisons, given our three hypotheses each tested for each model, resulting in six tests. The original *P* values were 0.001, 0.001, 0.001, 0.001, 0.001, and 0.003. After applying the BH adjustment, the *P* values were 0.006, 0.006, 0.006, 0.006, 0.006, and 0.003, all of which were below the 0.05 FDR threshold. This indicates that post-adjustment, all tests’ results remained statistically significant.

We also conduct the following exploratory analysis. Instead of comparing the LLM forecast after having been exposed to the human median to the LLM forecast before this exposure as preregistered, we compare this updated prediction to a simple average of the machine and human predictions as a naive benchmark using straightforward aggregation. This allows us to test whether the improvements the models make are due to genuinely understanding the need to appropriately update, as opposed to just providing an agreement-focused response. We find in paired *t* tests that for both GPT-4, at a Brier score of 0.13, *t*(92) = 2.583, *P* = .011; and Claude 2, at a Brier score of 0.14, *t*(92) = 3.530, *P* = .001; their updated forecasts are significantly less accurate than a simple average between the machine and the human median forecasts, which have an average Brier score of 0.12. This suggests that the updating itself is directionally correct but fails to improve upon a simple benchmark. In addition, recall that data collection for Study 2 was less controlled than for Study 1, as community median predictions for the tournament data were released at different points in time. Thus, comparisons of baseline accuracy between the two studies are not straightforwardly possible. On top of that, there were also differences in information present to the human forecasters, as well as differences in LLM prompts that further make any such comparison difficult.

## DISCUSSION

Our results show that LLM ensemble prediction capabilities can rival the gold standard of the human crowd tournament method. Previous results on single models (*28*, *46*) showed that LLMs not only underperformed compared to a human crowd in a probabilistic forecasting context but also failed to clear simple benchmarks. Others (*59*) found mixed results where—in the context of time-series forecasting—LLMs overperformed or underperformed relative to humans depending on the treatment condition; for more applications of LLMs in time-series forecasting, see (*60*–*62*). However, taking into account more sophisticated systems built on top of LLMs, such as combined retrieval and reasoning systems (*46*), human-level prediction accuracy may already be considered matched in some aspects. The approach presented here, a wisdom of the silicon crowd ensemble approach with LLMs, may be productively exploited in a variety of real-world contexts, as this aggregation approach remains simple to implement. Our finding opens the door for straightforward, practically applicable steps like forecast aggregation to increase current AI models’ forecasting ability—to predict future events in politics, economics, technology, and other real-world subjects—to a level that is statistically indistinguishable from the human crowd. This opens up a lot of directly applied work, given that LLM prediction capabilities can inform decision-makers and businesses in circumstances where accurate probabilistic forecasts are difficult or expensive to acquire. Furthermore, since both our finding and the finding of Halawi *et al*. (*46*) suggest that placing individual LLMs in advanced systems can increase their forecasting ability to a market-competitive level, it is natural to expect LLM predictions to be more widely applied across society in the near future.

We find a variety of human-like biases and prediction behaviors in our sample of LLMs. For example, we observe the presence of an acquiescence bias (*49*, *50*) in model predictions, in that our models’ predictions are more likely to be above 50%, despite the resolution rate of all questions being almost even. In addition, the model predictions also strongly favor round numbers like lay human forecasters often do. This suggests a potential avenue for further prediction capability improvements, as many of these human-like features are unlikely to be optimal and may be reducible via targeted prompting.

Moreover, we find that both the aggregate and the individual models were badly calibrated, with most models showing overconfidence, i.e., they assign higher probabilities to outcomes than is warranted by the empirical facts. Improving calibration is central to providing reliable predictions over the long run (*63*), and our results of acquiescence bias suggest that this may be an actionable area for future work focusing on improving model-level prediction capabilities.

In addition, even though it was not our primary research question, we were not able to provide evidence that the LLM aggregate outperformed the set of individual models that comprise it after adjusting for multiple comparisons, which may be explained by the comparatively low sample size that makes detection of smaller effects difficult. For more details on these limitations, the reader is directed to the “Limitations” section in Discussion, which contains a discussion of the design trade-offs. Qualitatively, though, we do find that the LLM aggregate has a numerically lower Brier score compared to 9 out of the 12 models. This provides some evidence in favor of the wisdom of the silicon crowd, though note that these comparisons are not statistically significant after adjustments. Similarly, our finding that the LLM crowd median (which is a median of the model medians) has a percentile rank of more than 50% on 61% of questions might also be evidence of a wisdom of the silicon crowd effect, though note again that this analysis was not preregistered. Future preregistered research may directly pick up the question of how many models are needed, what type of diversity is beneficial, and whether this can be simulated by a single larger model with higher temperatures.

In practical terms, a central upside of this LLM ensemble approach—compared to the human counterpart—is its ease of implementation. Running forecasting tournaments is an expensive and time-consuming process that relies on the presence of experienced and interested human forecasters. Running our ensemble approach is substantially cheaper. Depending on the number of queries, the total model count, and the token size of the input, the cost may be in the area of $1 per forecast. However, there are some unique challenges with LLM forecast elicitation that are generally not found in human forecasting tournaments. For example, we found that Alibaba Cloud’s Qwen-7B-Chat was substantially more likely to refuse forecasting on potentially controversial questions like conflict. While it does not seem likely that model behavior like this will be widespread, content restrictions like this may reduce diversity in a way that is not present in human forecasters who generally hold a quite heterogeneous set of views. However, LLM ensemble prediction approaches remain a scalable solution that may be applied in a variety of practical settings.

Our results from Study 2 that pertain to LLM updating behavior contribute to the broad literature on human-AI interactions (*64*, *65*). While previous work in the context of forecasting has looked at how LLMs can augment humans in improving prediction accuracy (*29*), this paper provides evidence for the reverse. Specifically, our results show that machine predictions can be improved substantially by the provision of human cognition output drawn from forecasting tournaments. This finding suggests, at first glance, that LLM reasoning is already advanced enough to properly exploit the informational value provided by human cognition output. However, our exploratory analyses find that this process is substantially less effective than simply averaging the two estimates, suggesting that single human-AI methods based on the reasoning capabilities of frontier models (in this case, GPT-4 and Claude 2) still underperform simple aggregations.

On the other hand, our findings that both frontier models (GPT-4 and Claude 2) respond as expected in their forecast updates—reducing their uncertainty when the human estimate lies within their prediction intervals, and updating in relation to the distance between their own point estimate and the human forecasts—match past theory and results pertaining to human forecasters (*66*). This overall suggests that the ability of these models to reason and act as expected—by past theory and results pertaining to human forecasters—depends on the type of task and benchmark applied. While this is not a massively strict test of their reasoning abilities—as alternative explanations of model behavior being explained by simple expectation-fulfilling remain—it does provide some evidence of such reasoning abilities.

Both studies reported in this paper test LLM capabilities in a context where it is not possible that any of the answers used to resolve the questions were part of the training data, as we queried the models in real-time alongside the human tournament. Because the correct answers to the questions were unknown at the time of data collection—even to the study authors—this provides an ideal evaluation criterion for LLM capabilities: one at which our LLM ensemble approach beat the naive baseline and was indistinguishable from the human crowd gold standard. This raises a host of additional research avenues and practical applications for LLM-powered prediction.

In conclusion, the present paper is among the first to show that current LLMs are not statistically different from human crowd competitive level of performance in forecasting future real-world events. We show this by applying the simple, practically applicable method of forecast aggregation. This replicates the human forecasting tournament’s wisdom of the crowd effect for LLMs: a phenomenon we call the wisdom of the silicon crowd. Our finding opens up a number of areas for further research as well as practical applications since the LLM ensemble approach is substantially cheaper and faster than data collection from human forecasters. Future research may aim to combine the ensemble approach with model and scaffolding progress, which may potentially result in even stronger capability gains in the domain of judgemental forecasting.

### Limitations

We want to explicitly point out a central limitation of our paper’s methods that we chose as part of our design. The main design trade-off was choosing between real-time forecasts and forecasts that resolved after the models’ knowledge cutoff and the present. We chose the former to accommodate real-time human forecasts as a comparison group, to avoid concerns like lookahead bias (*67*), to ensure that uncertainty about actual knowledge cutoff does not affect results, to have a high level of external validity, and to enable internet-connected models to participate. However, this approach brought with it a substantially smaller set of questions to be studied than might have been possible had we chosen the alternative approach to question selection. While we believe that our approach is, in general, a more stringent test of machine prediction capabilities, the lower sample size of questions (aggregated to one ensemble prediction per question) is a substantial limitation of our results, potentially making identification of small effects (such as differences between the aggregate and the individual models) difficult.
