# Supplementary material for: Wisdom of the silicon crowd: LLM ensemble prediction capabilities rival human crowd accuracy
Source: Sci Adv. 2024 Nov 8;10(45):eadp1528. doi: 10.1126/sciadv.adp1528 (PMC11800985; doi:10.1126/sciadv.adp1528)
Supplement: Supplementary file 1 — Tables S1 to S6 [file sciadv.adp1528_sm.pdf]

Science Advances
AAAS

Supplementary Materials for

Wisdom of the silicon crowd: LLM ensemble prediction capabilities rival human crowd accuracy

Philipp Schoenegger *et al.*

Corresponding author: Philipp Schoenegger, p.schoenegger@lse.ac.uk

Sci. Adv. **10**, eadp1528 (2024)
DOI: 10.1126/sciadv.adp1528

This PDF file includes:

Tables S1 to S6

Table S1: **Full list of questions.**

**Questions**
Will a nearly continuous human chain stretch across the length of the Forth and Clyde Canal on 14 October 2023?
Will Hamas lose control of Gaza before 2024?
Will Yahya Sinwar cease to act as Hamas Chief in the Gaza Strip before 2024?
Will Israel carry out and explicitly acknowledge a deadly attack on Iran before 2024?
Will the Conservatives hold on to their seat in the Mid Bedfordshire by-election?
Will it be determined that Israel was responsible for the attack on the Al-Ahli Baptist Hospital in Gaza City before 2024?
Will the Federal Funds Rate be raised before December 14, 2023?
Will Peter Bone MP be suspended from Parliament in 2023?
Will George Weah win re-election in the 2023 Liberian General Election?
Will India request that another Canadian diplomat be recalled before 2024?
Will New Delhi experience a ”Very Unhealthy” or worse air quality index on at least four of the seven days for the week starting October 29?
Will Mike Johnson remain Speaker until 2024?
Will there be an additional Russian IPO on the MICEX in 2023?
Will Donald Trump spend at least one hour confined in a jail cell before January 1, 2024?
Will the second Starship integrated flight test achieve liftoff before January 1, 2024?
Will Sarah Bernstein or Chetna Maroo win the 2023 Booker Prize?
Will Bitcoin reach $40,000 before January 1, 2024?
Will Volodymyr Zelenskyy visit Israel before 2024?
Will Delhi perform cloud seeding before December 1, 2023?
Will the MONUSCO UN peacekeeping mission to the Democratic Republic of the Congo be extended with a military personnel ceiling above 11,000 before January 1, 2024?
Will OpenAI report having $\geq$99% uptime for ChatGPT and the OpenAI API in December 2023?
Will the November 2023 Israel-Hamas humanitarian pause be extended?

Continued on next page

Table S1 – continued from previous page

**Questions**
Will a majority of voters approve Venezuela's referendum on incorporating Guayana Esequiba into Venezuela?
Will any additional Republican candidates for president drop out before 2024?
Will there be a white Christmas in at least 4 of these 9 large European cities in 2023?
Will the US Supreme Court issue a decision on hearing the case about presidential immunity before January 1, 2024?
Before 2024, will it be announced that either of the Harvard or MIT presidents will vacate their positions?
Will a major shipping company announce that they are resuming shipments through the Red Sea before 2024?
Will the ban on imports of Apple watches with blood oxygen sensors take effect before December 27, 2023?
Will there be a US military combat death in the Red Sea before 2024?
Will NASA re-establish communications with Voyager 1 before 1 Jan 2024?

Table S2: **Distributional Statistics for LLM Aggregate and Human Crowd.**

Statistic	LLM Aggregate	Human Crowd
Mean	0.198	0.190
Median	0.130	0.160
Standard Deviation	0.121	0.189
Variance	0.015	0.036
Minimum	0.040	0.000
Maximum	0.490	0.774
Range	0.450	0.774
Skewness	0.771	1.269
Kurtosis	-0.780	1.423
25th Percentile	0.122	0.031
50th Percentile (Median)	0.130	0.160
75th Percentile	0.360	0.265
Interquartile Range (IQR)	0.238	0.234

Table S3: **Comparison of each LLM's forecasting accuracy with that of the aggregate.**

Model	P-Value	Corrected P-Value
GPT-4	0.08	0.46
GPT-4 (with Bing)	0.23	0.49
GPT3.5-Turbo-Instruct	0.26	0.49
Claude 2	0.80	0.80
Solar-0-70B	0.59	0.78
Llama-2-70B	0.18	0.49
PaLM 2 (Chat-Bison@002)	0.33	0.49
Coral (Command)	0.02	0.23
Mistral-7B-Instruct	0.29	0.49
Bard (PaLM 2)	0.76	0.80
Falcon-180B	0.65	0.78
Qwen-7B-Chat	0.32	0.49

Table S4: **Percentile ranks of LLM median Brier scores.**

Question	Percentile Rank
Question 1	52.78
Question 2	50.00
Question 3	55.56
Question 4	51.52
Question 5	61.11
Question 6	50.00
Question 7	51.39
Question 8	50.00
Question 9	69.70
Question 10	54.55
Question 11	45.45
Question 12	56.06
Question 13	53.03
Question 14	55.56
Question 15	48.61
Question 16	50.00
Question 17	50.00
Question 18	53.03
Question 19	58.33
Question 20	48.48
Question 21	57.58
Question 22	51.85
Question 23	51.67
Question 24	50.00
Question 25	58.33
Question 26	50.00
Question 27	59.26
Question 28	46.67
Question 29	61.67
Question 30	50.00
Question 31	62.96

Table S5: **Accuracy by question type.**

Forecast #	LLM Brier Score	Human Brier Score	Delta	Classification
1	0.36	0.18	0.18	Politics
2	0.12	0.26	−0.14	Conflict
3	0.36	0.40	−0.04	Conflict
4	0.16	0.00	0.16	Conflict
5	0.36	0.25	0.11	Politics
6	0.18	0.00	0.18	Conflict
7	0.20	0.10	0.10	Economics
8	0.06	0.20	−0.14	Politics
9	0.36	0.36	0.00	Politics
10	0.36	0.12	0.24	Politics
11	0.06	0.05	0.01	Climate
12	0.12	0.02	0.10	Politics
13	0.12	0.27	−0.15	Economics
14	0.09	0.01	0.08	Law
15	0.12	0.16	−0.04	Technology
16	0.12	0.10	0.03	Literature
17	0.13	0.25	−0.12	Economics
18	0.25	0.16	0.09	Politics
19	0.49	0.35	0.14	Climate
20	0.12	0.42	−0.30	Conflict
21	0.04	0.00	0.04	Technology
22	0.12	0.36	−0.24	Conflict
23	0.12	0.01	0.12	Politics
24	0.14	0.25	−0.11	Politics
25	0.36	0.10	0.26	Climate
26	0.09	0.77	−0.68	Law
27	0.36	0.05	0.31	Education
28	0.12	0.64	−0.52	Economics
29	0.12	0.04	0.08	Law
30	0.14	0.01	0.13	Conflict
31	0.36	0.00	0.36	Technology

Average Count and Delta for each Classification: Climate ($n = 3$, $\Delta = 0.1400$); Conflict ($n = 7$, $\Delta = -0.0347$); Economics ($n = 4$, $\Delta = -0.1714$); Education ($n = 1$, $\Delta = 0.3116$); Law ($n = 3$, $\Delta = -0.1740$); Literature ($n = 1$, $\Delta = 0.0264$); Politics ($n = 9$, $\Delta = 0.0643$); Technology ($n = 3$, $\Delta = 0.1185$)

Table S6: **Percentile table of LLM forecasts.**

Probability	Frequency	Cumulative Frequency	Cumulative Percentage
0	3	3	0.30
1	20	23	2.28
2	0	23	2.28
3	0	23	2.28
4	0	23	2.28
5	3	26	2.58
6	0	26	2.58
7	0	26	2.58
8	0	26	2.58
9	0	26	2.58
10	5	31	3.08
11	0	31	3.08
12	1	32	3.18
13	0	32	3.18
14	0	32	3.18
15	19	51	5.06
16	1	52	5.16
17	1	53	5.26
18	1	54	5.36
19	0	54	5.36
20	23	77	7.65
21	3	80	7.94
22	0	80	7.94
23	0	80	7.94
24	0	80	7.94
25	12	92	9.14
26	0	92	9.14
27	0	92	9.14
28	6	98	9.73
29	1	99	9.83
30	34	133	13.21
31	0	133	13.21
32	1	134	13.31
33	0	134	13.31
34	1	135	13.41
35	44	179	17.78

Continued on next page

Table S6 – continued from previous page

Probability	Frequency	Cumulative Frequency	Cumulative Percentage
36	5	184	18.27
37	1	185	18.37
38	0	185	18.37
39	0	185	18.37
40	58	243	24.13
41	1	244	24.23
42	2	246	24.43
43	2	248	24.63
44	0	248	24.63
45	29	277	27.51
46	0	277	27.51
47	0	277	27.51
48	1	278	27.61
49	0	278	27.61
50	38	316	31.38
51	0	316	31.38
52	8	324	32.17
53	1	325	32.27
54	1	326	32.37
55	57	383	38.03
56	2	385	38.23
57	0	385	38.23
58	4	389	38.63
59	0	389	38.63
60	149	538	53.43
61	3	541	53.72
62	5	546	54.22
63	1	547	54.32
64	0	547	54.32
65	121	668	66.34
66	1	669	66.43
67	1	670	66.53
68	3	673	66.83
69	0	673	66.83
70	132	805	79.94
71	0	805	79.94
72	6	811	80.54

Continued on next page

Table S6 – continued from previous page

Probability	Frequency	Cumulative Frequency	Cumulative Percentage
73	3	814	80.83
74	0	814	80.83
75	52	866	86.00
76	8	874	86.79
77	0	874	86.79
78	5	879	87.29
79	2	881	87.49
80	49	930	92.35
81	1	931	92.45
82	1	932	92.55
83	0	932	92.55
84	0	932	92.55
85	18	950	94.34
86	1	951	94.44
87	0	951	94.44
88	2	953	94.64
89	0	953	94.64
90	16	969	96.23
91	0	969	96.23
92	1	970	96.33
93	0	970	96.33
94	3	973	96.62
95	8	981	97.42
96	2	983	97.62
97	1	984	97.72
98	0	984	97.72
99	22	1006	99.90
100	1	1007	100.00
